# Retraction: Exogenous application of moringa leaf extract improves growth, biochemical attributes, and productivity of late-sown quinoa

**DOI:** 10.1371/journal.pone.0272392

**Published:** 2022-08-17

**Authors:** The PLOS ONE Editors

The *PLOS ONE* Editors retract this article [1, 2] because it was identified as one of a series of submissions for which we have concerns about authorship, competing interests, and peer review. We regret that the issues were not addressed prior to the article’s publication.

SK, DI, SI, ZH, and ATKZ did not agree with the retraction. NR, AW, AB, JA, MSA, and MRAG either did not respond directly or could not be reached.
